# Clinical Use of Progestins and Their Mechanisms of Action: Present and Future (Review)

**DOI:** 10.17691/stm2021.13.1.11

**Published:** 2021-02-28

**Authors:** T.A. Fedotcheva

**Affiliations:** Senior Researcher, Research Laboratory of Molecular Pharmacology, Pirogov Russian National Research Medical University, 1 Ostrovitianova St., Moscow, 117997, Russia

**Keywords:** progesterone, megestrol acetate, medroxyprogesterone acetate, gestagens, progesterone receptors

## Abstract

This review summarizes the current opinions on the mechanisms of action of nuclear, mitochondrial, and membrane progesterone receptors. The main aspects of the pharmacological action of progestins have been studied. Data on the clinical use of gestagens by nosological groups are presented. Particular attention is paid to progesterone, megestrol acetate, medroxyprogesterone acetate due to broadening of their spectrum of action. The possibilities of using gestagens as neuroprotectors, immunomodulators, and chemosensitizers are considered.

## Introduction

Progesterone is a natural endogenous steroid sex hormone secreted by the ovaries. It interacts with its specific receptors in the reproductive tract, the mammary gland, and the central nervous system. Progesterone and progestins have been used for decades for contraception, maintenance of pregnancy with threatened miscarriage, for symptomatic therapy of postmenopause, secondary amenorrhea, abnormal uterine bleeding [1, 2]. In the last 30 years, progestins have been used for treatment of endometriosis and hormone-sensitive tumors, and during the last 15 years — in ART (assisted reproductive technologies) procedures [3]. Clinical efficacy of some gestagens has been confirmed in management of cachexia and anorexia in cancer patients and AIDS patients [4]. Owing to the discovery of previously unknown targets for progestin action (membrane-associated progesterone receptors; xenobiotic transport proteins, mitochondrial pores; checkpoint signaling pathway proteins), new aspects of clinical use of progesterone and its synthetic analogues have emerged.

## Mechanism of progestin (gestagen) action

**Figure 1:**
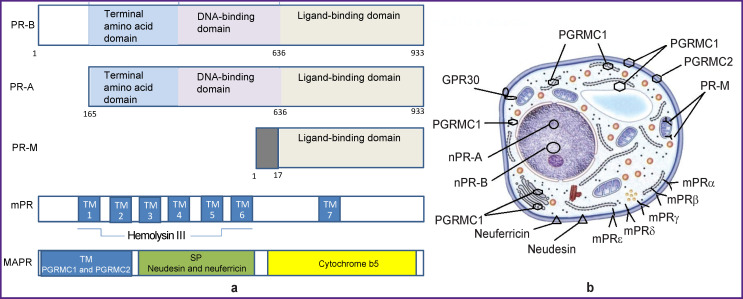
Structure and localization of progesterone receptors: (a) protein domains of nuclear (nPR-A, nPR-B), mitochondrial (PR-M), membrane (mPR) and membrane-associated progesterone receptors (MAPR); (b) localization of progesterone receptors in the cell: nPR-A and nPR-B are localized in the nucleus, PGRMC1 — in the cell membrane, endoplasmic reticulum, the Golgi apparatus, in the cytoplasm, PR-M — in mitochondria, other membrane receptors and MAPR — in the cell membrane. Here: TM — transmembrane domain in PGRMC1 and PGRMC2; SP — signal peptide in neudesin and neuferricin; GPR30 — G-protein-coupled receptor

The mechanism of progestin action is realized through nuclear, mitochondrial, and membrane progesterone receptors (Figure 1).

*Nuclear progesterone receptors* (nPR) are represented by isoforms A and B (nPR-A and nPR-B), which are transcription products of one gene resulting from the action of different promoters. When gestagen binds to nuclear receptors (transcription factors), it is accompanied by genomic effects that develop over several hours and days, leading to specific physiological and morphological changes in target organs, which is the classic progestin action [5].

Progesterone also induces rapid (minutes, hours) stimulation of cellular signaling cascades through membrane progesterone receptors (mPR) and membrane-associated progesterone receptors (MAPR) which are two different types of *membrane receptors* [6].

Membrane receptors of the first type are adipoQ receptors (PAQR): mPRα (PAQR7), mPRβ (PAQR8), mPRγ (PAQR5) are associated with inhibitory G-proteins, they reduce the intracellular concentration of cAMP; mPRδ (PAQR6) and mPRε (PAQR9), on the contrary, increase cAMP [7]. Receptor mPRα is expressed mainly in the human reproductive organs, mPRβ — in the central nervous system, while mPRγ — in the kidneys and intestines. The latest identified receptors mPRδ and mPRε are still understudied, but their neuroprotective function and localization in the central nervous system are already obvious. For example, brain biopsies from men and women who died unexpectedly at the age of 16– 65 years showed that mPRδ and mPRε were present in all brain structures; in high-affinity cell models, they bind neurosteroids dehydroepiandrosterone, pregnanolone, pregnenolone, allopregnanolone and produce their anti-apoptotic effect [8].

Membrane receptors of the second type are members of b5-like heme/steroid-binding protein family: the membrane component of the 1^st^ and 2^nd^ progesterone receptors (PGRMC1 and PGRMC2); neudesin neurotrophic factor (NENF) and neuferricin (CYP5D2) with transmembrane localization [9]. They are found in the forebrain structures where progesterone is responsible for neuroendocrine regulation and other non-genomic effects: membrane transport, steroidogenesis, iron homeostasis, and heme transport due to regulation of hepcidin expression and ferrochelatase activity, lipid transport, migration of nerve axons, synaptic function, and antiapoptotic action. PGRMC1 has been studied best of all as a multifunctional protein with a heme-binding site, providing transfer of partner proteins (estradiol receptors, growth factors, cytochromes) from intracellular depots to the point of meeting with a specific ligand [5]. Localization of PGRMC1 can be cytoplasmic, nuclear/nucleolar, mitochondrial, in the endoplasmic reticulum, in cytoplasmic vesicles, or extracellular (Figure 1 (a)). PGRMC1 is involved in cell cycle processes at the G1 checkpoint during mitosis, while overexpression of PGRMC1 is associated with adverse prognosis in many types of cancer [10]. PGRMC2 is structurally similar to PGRMC1, although there are differences in the N-terminal transmembrane domain. PGRMC2 is not well understood, but it is known to participate in cell cycle processes and to inhibit cell migration during tumor invasion [9]. Neudesin exhibits neurotrophic activity by heme binding, which is then mediated by mitogen-activated protein (MAP) and phosphatidylinositol-3-kinase (PI3K). Neuferricin (cytochrome b5 domain) was discovered via a homology-based search of the CYB5-like heme/steroid-binding domain of neudesin. A rare human condition of cytochrome b5 deficiency causes disorders of sexual development and appearance of hermaphroditism signs [11].

The latest discovered non-nuclear progesterone receptor is *mitochondrial progesterone receptor* (PR-M). It is a truncated version of nPR localized in mitochondria (Figure 1 (b)) [12]. It is involved in direct ligand-dependent regulation of mitochondrial functions; increased production of cellular energy when needed — for example, during pregnancy. Investigation of the role of the mitochondrial receptor in various cellular processes is just beginning. There are many assumptions concerning its functions — increased proliferation and viability of lobular-alveolar cells of the mammary gland and myometrial cells, increased synthesis of myofibrils in the myocardium and myometrium. It is also necessary to consider the role it plays in increasing the rate of cellular metabolism by inducing ATP production in mitochondria [6]. Progesterone-dependent increase in basal body temperature has traditionally been associated with its major influence on the hypothalamus, but identification of PR-M casts doubt on this fact.

Non-classical mPR and MAPR are believed to evolve first in the ancient bilateria, while classical nuclear progesterone receptors appeared later in vertebrates [13].

## Pharmacological action of gestagens

The main pharmacological effects of progesterone, exerted mainly by nuclear receptors, are the following:

endometrial secretory transformation;

formation of thick and viscous cervical mucus;

increased basal temperature;

reduced activity of genital tract and uterine smooth muscles (tocolytic effect);

activation of mammary gland secretory acini growth and induction of lactation;

protein lipase stimulation; increased fat stores;

basal and induced insulin levels and glucose utilization rate;

liver glycogen accumulation; aldosterone production;

hypoazotemia and azoturia;

increased (in small doses) or suppressed (in large doses) gonadotropic hormone production in the pituitary gland.

Pharmacological effects of progestins (progesterone analogues) are usually assessed during a number of preclinical studies in animals, namely:

determination of affinity for progesterone receptors;

assessment of gestagenic activity using endometrial transformation test, pregnancy maintenance test, and ovulation inhibition test;

assessment of androgenic activity by measuring the weight of the prostate or levator ani muscle;

measuring anti-androgenic activity or evaluating feminizing activity in male rats;

analysis of glucocorticoid and antimineralocorticoid properties [14].

The most important structural and functional role in the mechanism of gestagen action is played by substituents at the carbon atoms C3 and C17 of the cyclopentaneperhydrophenanthrene ring — the steroid nucleus. It is well known that even small structural differences in steroid molecules cause significant differences in their clinical effects on target organs and on the risk of developing cardiovascular diseases. Specifically, natural progesterone and some of its derivatives, for example, drospirenone, have potent anti-mineralocorticoid activity and a beneficial effect on blood pressure; dienogest has no androgenic effect and, therefore, no negative influence on lipid and carbohydrate metabolism and directly on endothelial cells. Investigation on mice fed a diet high in fat and drospirenone showed no increase in body weight or adipose tissue mass or changes in the level of glucose tolerance due to drospirenone antagonism to mineralocorticoid receptors controlling adipocyte function [15]. Gestagens lacking a keto group at the C3 carbon atom (mepregenol derivatives — Acetomepregenol, gestobutanoil) are unable to bind to androgenic and mineralocorticoid receptors, therefore they produce no side mineralocorticoid, androgenic, virilization effects [16, 17].

## Clinical use of gestagens

The dosage forms of gestagens existing in the Russian pharmaceutical market and indications for their use are presented in the Table.

Gestagens are successfully used for contraception, pregnancy maintenance, and secondary amenorrhea. They are the front-line therapy for endometrial hyperplasia and cancer [18–20]. Gestagens are used in hormone replacement therapy (HRT) to prevent osteoporosis and in ART procedures [21].

A new aspect of clinical use of gestagens is treatment of cachexia and anorexia with megestrol acetate. It promotes weight gain, increased appetite, but the mechanism of this effect remains unclear [22]. The effect of megestrol acetate on anorexia and weight gain might be associated with inhibition of pro-inflammatory cytokine production (IL-1, IL-6, various TNFs) and stimulation of the hypothalamus with neuropeptide Y [23]. In 1993, the Food and Drug Administration (FDA) approved its use for treatment of anorexia, cachexia, or unexplained weight loss in patients with acquired immunodeficiency syndrome (AIDS). The advantage of this gestagen is the absence of general toxic, mutagenic or carcinogenic properties. Therefore, treatment of endometrial cancer with Megace based on megestrol acetate allows young patients to maintain reproductive function [24].

**Figure 2:**
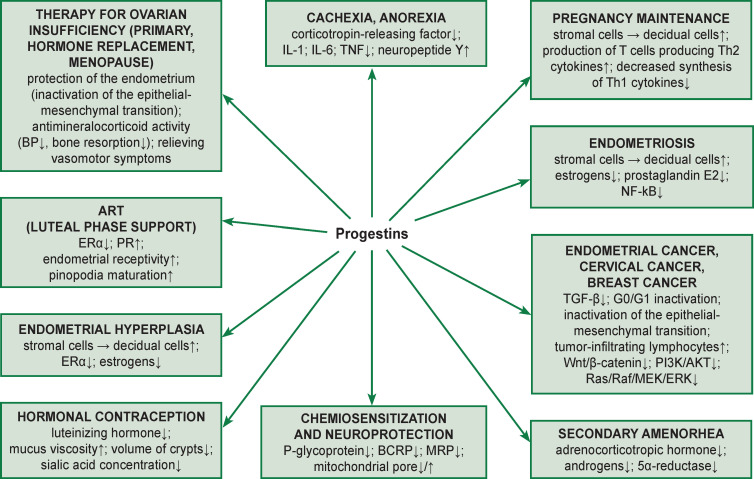
The main aspects of clinical use of gestagens with the mechanism of action

The main aspects of clinical use of progestins and their mechanism of action are shown in Figure 2.

### Gestagens in contraception and pregnancy maintenance

Progesterone plays an important role in the onset and development of pregnancy (“pregnancy hormone”) due to various mechanisms. They include modulation of the mother’s immune response and suppression of the inflammatory response, reduction of uterine contractility (adequate progesterone concentrations in the myometrium can counteract the stimulating activity of prostaglandin and oxytocin), improvement of blood circulation in the uteroplacental system and luteal phase support (progesterone promotes penetration of extravillous trophoblasts into decidual cells by inhibiting apoptosis of these trophoblasts [25].

In the 50–80s of the last century, the main indication for administration of progestins was pregnancy maintenance. Today, the gold standard is the use of dydrogesterone (Duphaston) and progesterone (Utrogestan), their efficacy has been proven by many clinical studies [26–30].

Later, contraceptives based on gestagens appeared in the pharmaceutical market. The greatest variety of trade names and dosage forms has been created specifically for these purposes. Millions of women use combined oral contraceptives (COCs) because they are highly effective in preventing pregnancy. However, COCs appear to be unsuitable for some women because of cardiovascular risks associated with their use. In addition, they are capable of producing side effects due to the presence of estrogens in their composition: headache, nausea, weight gain, breast tension, and others. Unlike the widely used COCs, there are progestogen-only pills taken continuously without any breaks between packets (mini-pills) and various devices — coils and patches producing small amounts of gestagen daily. Progestagen-only formulations (progestin-only contraceptives — POCs) are an acceptable option for women in whom estrogens are contraindicated. Among the variety of mini-pills in the US pharmaceutical market, there are POCs containing norethindrone, while tablets with levonorgestrel or desogestrel are available on the international pharmaceutical market [31]. The drug Lactinette based on desogestrel is the leader of mini-pills market in the Russian Federation.

**Table:** Dosage forms and indications for the use of progestins in Russia

ICD-10/Nosology	Gestagen	Trade name	Dosage form
O20.0 Threatened abortion	Dydrogesterone	Duphaston®	Tablets, 10 mg
	Micronized progesterone	Utrogestan®	Capsules, 100 and 200 mg
	Hydroxyprogesterone caproate	Oxyprogesterone capronate®	Solution for injection in oil 12.5% in ampoules
	*Allylestrenol*	*Turinal®*	Tablets, 5 mg
	*Acetomepregenol*	*Acetomepregenol®*	Tablets, 0.5 mg
Z30 Monitoring the use of contraceptives	Desogestrel	Lactinette®	Tablets, 0.075 mg
	*Desogestrel*	*MODELLE® MAM*	Tablets, 0.075 mg
	Levonorgestrel	Microlut®	Tablets, 0.03 mg
	Levonorgestrel	Mirena®	Intrauterine therapy system with guide, 52 mg
	Levonorgestrel	MODELLE® 911	Tablets, 1.5 mg
	Levonorgestrel	Norplant®	Subcutaneous implantable capsules containing 36 mg of levonorgestrel
	Levonorgestrel	Postinor®	Tablets, 0.75 mg
	Levonorgestrel	Escapelle®	Tablets, 1.5 mg
	*Levonorgestrel*	*Eskinor-F*	Tablets, 0.75 mg
	Lynestrenol	Exluton®	Tablets, 0.5 mg
	Lynestrenol	Orgametril®	Tablets, 5 mg
	*Acetomepregenol*	*Acetomepregenol®*	Tablets, 0.5 mg
	Norethisterone	Norkolut®	Tablets, 5 mg
	Etonogestrel	Implanon NXT®	Implant, 68 mg
N91 Suppressed menstruation, scanty and infrequent menstruation N91.1 Secondary amenorrhea	Progesterone	Krinon®	Vaginal gel, 90 mg/dose, 15 pcs.
N80.0 Endometriosis	Dydrogesterone	Duphaston®	Tablets, 10 mg
	Dienogest	Visanne®	Tablets, 2 mg
	Norethisterone	Norkolut®	Tablets, 5 mg
	*Norethisterone*	*Primolut®-Nor*	Tablets, 5 and 10 mg
N85.0 Glandular endometrial hyperplasia	Levonorgestrel	Mirena®	Intrauterine therapy system with guide, 52 mg
C54 Malignant neoplasm of the uterine body	*Gestonorone caproate*	*Depostat®*	1 ml oil solution for injection containing 100 mg of Gestonorone caproate
	Hydroxyprogesterone caproate	Oxyprogesterone capronate®	Solution for injection in oil 12.5% in ampoules
	Linestrenol	Orgametril®	Tablets, 5 mg
C50 Malignant neoplasm of breast	*Gestonorone caproate*	*Depostat®*	1 ml oil solution for injection containing 100 mg of Gestonorone caproate
	Medroxyprogesterone	Provera®	Tablets, 100 and 500 mg
C53 Malignant cervical neoplasm	Hydroxyprogesterone caproate	Oxyprogesterone capronate®	Solution in oil 12.5% in ampoules
C54.1 Malignant endometrial neoplasm	Medroxyprogesterone	Provera®	Tablets, 100 and 500 mg
Z31.1 Assisted reproductive technologies	Progesterone	Krinon®	Vaginal gel, 90 mg/dose, 15 pcs.
	Progesterone	Utrogestan®	Capsules, 100 and 200 mg

Note: italics indicate drugs temporarily absent on the Russian pharmaceutical market.

The contraceptive action of progestins is mediated through the following mechanisms [32]:

1. Inhibiting effect on the secretion of gonadotropic hormones by the pituitary gland (especially luteinizing hormone) and, as a consequence, inhibition of ovulation (depends on the dose of gestagen in the tablet).
2. Increased viscosity of cervical mucus. Progestins reduce crypt volume, thicken cervical mucus, reduce sialic acid content in the mucus and sperm activity, they narrow the cervical canal, thereby preventing penetration of sperm and some microorganisms into the cervical canal, uterus, and fallopian tubes.
3. Specific effect on the endometrium. Progestins suppress mitotic activity of the endometrium, promoting its premature secretory transformation. Long-term use of progestagens under conditions of anovulation causes hypotrophy and atrophy of the endometrium, which prevents implantation of the fertilized ovum.
4. Decrease in the contractile activity of the fallopian tubes by reducing the contractility and excitability threshold of smooth muscle cells of their walls.

One of the latest developments is Implanon NXT, a long-acting (3 years) subcutaneous radiopaque hormonal contraceptive with etonogestrel [33, 34]. It is distinguished by a rapid onset of action and a rapid return of fertility after withdrawal, the absence of mineralocorticoid activity. This drug is easy to use, its efficacy does not depend on the correct use, which is very important, because unplanned pregnancy often occurs due to inappropriate usage of contraceptives [35, 36].

In recent years, there has been a tendency towards an increase in the number of women who have made their choice in favor of LARC-methods of contraception (long-acting reversible contraception) [37, 38].

### Endometriosis

Success of the disease treatment depends on individually selected drug therapy based on the molecular genetic characterization of the patient’s endometrioid tissue [39]. The main molecular mechanisms of gestagen action on the pathogenetic components of endometriosis are as follows: 1) gestagens inactivate the hypothalamic-pituitary-ovarian axis of estrogen synthesis; 2) competitively bind to estrogen receptors, providing an anti-estrogenic effect; 3) inhibit prostaglandin E_2_ synthesis; 4) inhibit the enzyme aromatase (CYP19); 5) activate the HSD17B2 enzyme; 6) cause direct inhibition of nuclear factor-kappa B (NF-kB) playing a key role in the processes of inflammation and neoangiogenesis; 7) have a direct effect on endometriotic foci, causing differentiation of stromal cells (decidualization) and secretory transformation of endometrial epithelial cells, which results in endometrial atrophy.

Currently, only three gestagens are recommended for the treatment of endometriosis: medroxyprogesterone acetate (MPA), dienogest, and dydrogesterone. Of these drugs, only dienogest has direct indications for the treatment of endometriosis in Russia; in other countries, both MPA and dydrogesterone are used [16, 40, 41].

There is a novel domestic development at the stage of clinical trials — gestagen gestobutanoil in tablet dosage form, which is equal to foreign dienogest in efficacy. It was revealed on experimental models of endometriosis that the drug has two metabolites, one of which is megestrol acetate circulating in the blood for a long time. This property of the drug can ensure its prolonged gestagenic action [17, 42–46].

### Endometrial hyperplasia

Endometrial hyperplasia (EH) is defined by some authors as morphological and biological changes in the endometrium resulting from prolonged stimulation with estrogens against the background of progesterone deficiency [47]. EH is a precancerous condition of the endometrium. Cytological atypia with expression of such markers as pyruvate kinase M2, β-catechin, and the presence of MELF (microcystic, elongated, fragmented glands) regions [48–50] seem to be the most important factors in transformation of hyperplasia into carcinoma. The main goal in treating women with EH is not only to reduce the frequency and intensity of abnormal uterine bleeding, but also to prevent the endometrium from transformation into a tumor. The mechanism of the protective effect of progestins on the endometrium lies in the fact that they transfer the endometrium from the proliferative phase to the secretory phase, causing the so-called secretory transformation of the endometrium. The second mechanism is anti-estrogenic, i.e. there is a decrease in the mitotic effect of estrogens on the endometrium (see Figure 2).

Levonorgestrel in the form of the Mirena intrauterine system is recommended as the front-line drug therapy in Russia and abroad [51]. Levonorgestrel does not reduce the volume of the ovaries and ovarian reserve and, compared to COCs, slightly reduces the concentration of anti-Müllerian hormone [52], which allows young women with EH to maintain reproductive function.

The most important strategy in the treatment of EH is controlling the potential of endometrial malignancy, therefore it is necessary to perform regular immunohistochemical studies, in particular, to determine such markers as PTEN, p53, β-catechin, Bcl-2, COX-2, p27, p21, MLH-1, -2 and -6, survivin, p16, and also expression of estrogen and progesterone receptors (ER-α, ER-β, nPR-A, nPR-B) [53].

### Endometrial cancer and cervical cancer

Endometrial cancer (EC) is the most common type of neoplasm in gynecology; every year it is detected with increasing frequency [54]. The use of COCs is considered to be a protective, prophylactic factor reducing the risk of EC development, the residual protective effect lasting up to 30 years after discontinuation of the drug [55].

The first-line drug therapy for EC is the use of classical cytostatics (doxorubicin, cisplatin, paclitaxel, and others) and hormone therapy (MPA, megestrol acetate, tamoxifen) [56]. Treatment with progestins is quite effective: the response rate ranges from 11 to 56%, depending on EC type (the greatest efficacy is achieved with endometrioid EC (type I)). Targeted therapy for EC leads to no good results: as evidenced by the analysis of clinical studies, the response rate does not exceed 33% with the use of various drugs [54].

Clinical studies have repeatedly confirmed MPA efficacy in EC treatment, especially as neoadjuvant therapy [57–59]. MPA activates estrogen stress, blocks signal transduction from the estrogen receptor by binding to nPR-B, and increases expression of the enhancer protein CHOP, which might be one of the molecular mechanisms underlying the antitumor effect of MPA in EC. In cells containing nPR-B (nPR-B+), long non-coding RNAs (lncRNAs), Inc-CETP-3, are involved in this process. Recently, there have been identified differentially expressed mRNAs and lncRNAs involved in signaling cascades of carcinogenesis, which can become a target for anticancer drugs and, in particular, for gestagens [60].

The optimal regimen for the use of gestagens in EC is daily oral intake of 200–400 mg of MPA (Provera) or 160 mg of megestrol acetate (Megace). The effect of progestin therapy begins to manifest itself no earlier than 8–12 weeks after the start date of drug administration [61].

The use of gestagens in breast cancer and cervical cancer (CC) is limited because there is insufficient information about their effects on these types of cancer. Interim results of a large (1000 patients) clinical trial, Primary Progesterone Therapy for Operable Breast Cancer, show that progesterone therapy significantly increases relapse-free survival in patients with lymph node involvement, but does not affect this parameter in patients with primary breast cancer without lymph node involvement [62].

Despite the fact that hydroxyprogesterone caproate has indications for the treatment of CC, according to ICD-10 C53 “Malignant Cervical Neoplasm”, hormone therapy is currently not recommended for treatment of this disease in the Russian Federation [63]. Hormone therapy is not used by the European communities of oncologists either [64]. However, a 0.25% solution of oxyprogesterone capronate (17-OPC) is recommended according to an individual therapeutic regimen only in glandular forms of CC.

The mechanism of the cytotoxic action of progesterone (and its analogues) consists of inducing the mitochondrial apoptosis and preventing cell cycle transition from the G1 to S phase [65]. It has been confirmed that regulation of expression and activity of the transient receptor potential melastatin-subfamily member 7 (TRPM7), which triggers acidotoxic necrosis in CC cells, plays the role in the direct cytotoxic effect of progesterone. Progesterone inhibits TRPM7 expression, thereby switching acidotoxic necrosis of tumor cells to apoptosis [66]. A number of studies on HeLa cells have demonstrated the cytotoxic effect not only of progesterone, but also of its synthetic derivatives, the cytotoxic effect being observed both upon stimulation with estradiol and without stimulation [67, 68].

The challenges of using gestagens in EC are associated with improvement of administration regimens based on the individual status of the patient and inclusion of other drugs, in particular metformin, in the treatment regimens. The antitumor mechanism of metformin action is poorly understood, but it appears to be related to the mTOR signaling pathway. Metformin is a biguanide widely used in the treatment of type 2 diabetes. Analysis of several recent epidemiological studies [19, 69, 70] has shown that metformin reduces the risk of cancer development and mortality in diabetic patients with endometrial hyperplasia and carcinoma.

Since the incidence of EC in young patients often correlates with obesity, insulin resistance, and impaired glucose metabolism (84, 83, and 78%, respectively), these provoking factors are a promising target for treatment and prevention of this disease with metformin [71–73].

### Hormone replacement therapy

The role of gestagens in HRT mainly involves protecting the endometrium from malignization, exerting antimineralcorticoid action, preventing osteoporosis, and relieving psychoemotional and vasomotor symptoms [74].

Professor Jerilynn C. Prior of the University of British Columbia (Vancouver, Canada) is an expert in clinical research on progesterone in HRT. She found that in HRT progesterone effectively reduces vasomotor symptoms in perimenopausal women, prevents osteoporosis, normalizes sleep, and eliminates anxiety [75]. The beneficial effect of progesterone on physiological functions, psychoemotional state, and life expectancy has been shown even in transgender women [76].

Besides, progestins are effective in treatment of secondary amenorrhea: vaginal progesterone — Crainon gel — is the drug of choice [77].

### Assisted reproductive technologies

Assisted reproductive technologies are increasingly used in modern medicine. The protocols of *in vitro* fertilization (IVF), artificial insemination, and transfer of cryo-preserved embryo transfer are constantly being improved as evidenced by a number of initiated and completed clinical studies [78, 79].

Most ART cycles are accompanied by formation of luteal phase insufficiency. Therefore, gestagen drugs are widely used in infertile women during ART when superovulation is stimulated or cryo-preserved embryo transfer program requiring gestagen therapy is performed [80]. Progesterone reduces ER-α levels, increases expression of progesterone receptors, thereby increasing sensitivity of the endometrium to progesterone and endometrial receptivity. In contrast to the proliferative effect of estrogen, progesterone action promotes differentiation of endometrial tissue inactivating estradiol through stromal progesterone receptors, emergence and maturation of pinopodia, ensures preparation of the endometrium for embryo implantation [81]. Progestins increase not only endometrial receptivity but also its thickness, which is an important factor in ensuring successful embryo implantation [82, 83].

Thus, ART is a relatively new nosological group for the clinical use of gestagens and its efficacy in improving fertility is uncontroversial.

## Non-classical pharmacological effects of progestins — a platform for new clinical trials

The future of using gestagens in clinical practice is determined by the discovery of new targets for progesterone action on the immune system, the cardiovascular system, the central nervous system, and its specific, unique effect on drug metabolism, involving cytochrome systems, enzymes, and drug resistance proteins such as P-glycoprotein, BCRP protein, MRP proteins, and others.

***Immunomodulatory effects of progestins*** are mainly associated with suppression of excessive immune response: inhibition of lymphocyte activation and proliferation in response to mitogenic and immune stimuli, which plays an important role in pregnancy maintenance as cytokine-mediated immunological reactions cause 40–60% of all recurrent idiopathic spontaneous miscarriages.

It has been recently revealed that maternal immune tolerance towards the fetus is a key factor contributing to its development. Successfully progressing pregnancy is accompanied by suppressed activity of type 1 T-helpers (Th1) and increased Th2 activity. For instance, increased levels of Th1-cytokines, IL-2, and interferon-γ and decreased levels of Th2-cytokines and IL-10 were observed in women with recurrent spontaneous miscarriage [84].

Expression of cytokines and chemokines IL-6, IL-8, CCL2, CXCL1, CXCL2 in endothelial cells is specifically inhibited by progesterone, which indicates that progestins are anti-inflammatory agents in the endothelium with the potential to suppress the transport of immune cells into tissues [85]. The anti-inflammatory effect of progesterone was confirmed in the study by VanLandingham et al. Progesterone stimulates expression of membrane protein CD55 in the rat brain, a potent inhibitor of the complement system convertases, leading to inflammatory cascade inhibition [86].

Specific regulation of cytokine synthesis by progesterone might prove crucial in suppressing the cytokine storm in sepsis and viral diseases such as COVID-19. One of the main reasons for the death of patients infected with the SARS-CoV-2 coronavirus is the development of excessive immune response, a cytokine storm, leading to severe damage to vital organs. Pharmacological correction of the cytokine storm in severe forms of COVID-19 will likely save lives of the infected patients and achieve stabilization and improvement in patient conditions.

The drugs proposed for pharmacological correction of the cytokine storm during SARS-CoV-2 infection are glucocorticoids known for their prominent anti-inflammatory activity. However, their simultaneous strong immunosuppressive effect may limit the use of glucocorticoids [87]. Other drugs used for this purpose (the antimalarial agent hydroxychloroquinone and the cytostatic antitumor agent etoposide) also have serious toxic side effects [88, 89].

Hence, it seems appropriate to estimate the possibilities of gestagen derivatives (they are well tolerated and act as selective immunomodulators) for the immune correction of the cytokine storm. It has been shown that some progesterone derivatives possess prominent anti-inflammatory and immunomodulatory activity due to their unique action associated with inhibition of mitochondrial proton pumps, regulation of intracellular pH, lysosomal enzyme activity, toll-like receptors of immune cells, and others [90–92].

### Neuroprotective effects of progestins

Progesterone is also a neurosteroid synthesized in neurons and glial cells. Progesterone metabolites — 5α-dihydroprogesterone and 3α, 5α-tetrahydroprogesterone (allopregnanolone) — have a neuroprotective effect in traumatic brain injury; spinal cord injuries; in ischemic damage to the brain and spinal cord; in neurodegenerative diseases, including cerebral atherosclerosis, Parkinson’s and Alzheimer’s disease. Progesterone reduces cerebral edema, inflammation, pro-oxidant activity of metabolites, restores mitochondrial membrane potential, regulates hemostatic proteins and promotes survival of newly formed neurons, participates in maturation and myelination of neurons. Allopregnanolone is GABA_A_ receptor positive allosteric modulator (gamma-aminobutyric acid receptors are chloride ion channels). As a result, progesterone and allopregnanolone affect synergistically many functions in the brain via progestin and GABA_A_ receptors [93].

The human central nervous system was found to have all five types of membrane receptors with some differences in the level of their expression and distribution, as well as MAPR — PGRMC1, PGRMC2, neudesin, and neuferricin. Their functions are not well defined, but there is evidence that they are involved in the synthesis and transfer of heme, suppression of induced cell death, and apoptosis in cells [9].

Other important functions of progesterone in the central nervous system are modulation of the activity of neurons secreting gonadotropin-releasing factor, regulation of luteinizing hormone secretion, and regulation of monoamine (dopamine and serotonin) metabolism [94].

**The effect of gestagens on carcinogenesis** is associated with proliferation, apoptosis, and epithelial-mesenchymal transition of EC, breast cancer, CC in tumor cells. The main mechanisms of antitumor activity of gestagens are determined by a decrease in progesterone receptors nPR-A and estrogenic ER-β, induction of apoptosis by stimulating production of reactive oxygen species in high doses [95]. Megestrol acetate and MPA in high doses also have a direct receptor-mediated cytotoxic effect on tumor cells, in low doses, they reduce estrogen secretion and synthesis, reduce production of growth hormones, and increase production of tumor necrosis factors. Induction of the Fas/FasL system components by progesterone increases sensitivity of target cells to apoptotic signals. Progesterone can act as a regulator of alternative splicing of TGF-β receptor gene. Besides, progestins regulate the activity of target cell enzymes involved in transmission of cell death signal, modulating cell response to the apoptotic signal [96].

Progesterone and its synthetic analogs can trigger the mitochondrial pathway of apoptosis. Mitochondrial membrane potential drop leads to formation of reactive oxygen species and release of apoptogenic factors (release of cytochrome C and activation of caspases), serving as a signal for activation of the final (effector) stage of apoptosis. Existing models of mitochondrial implementation of the apoptotic program take into account activity of regulatory protein family Bcl-2, disrupting mitochondrial homeostasis independently or through other mitochondrial proteins (adenine nucleotide carriers (ANT), voltage-dependent anion channels (VDAC)) [95]. It has been established that steroid hormones, in particular progesterone, produce extragenomic effects on mitochondrial processes aimed at inducing apoptosis due to the influence on ionic fluxes Ca^2+^ and K^+^, on the one hand, and expression of mitochondrial apoptosis-inducing factors (family Bcl-2) on the other hand [97]. Our latest data point to the existence of membrane progesterone receptors whose localization, including that on the mitochondrial membrane, can determine the effect of gestagens on the transcription of mitochondrial genes and metabolic processes in mitochondria [98].

Progesterone is involved in regulation of four major signaling pathways for carcinogenesis: PI3K/AKT, Ras/ Raf/MEK/ERK, WNT/β-catenin, and VEGF. The PI3K/ AKT pathway activates the transforming growth factor beta (TGF-β)-mediated endothelial-to-mesenchymal transition (EMT). Progesterone inhibits EMT partly owing to its inhibitory effect on TGF-β. *In vitro*, progesterone inhibits TGF-β signaling 72 h after treatment of Ishikawa endometrial cancer cells and effectively suppresses the viability and invasion of endometrial cancer cells with increased E-cadherin expression [99].

In addition to inhibiting EMT, progesterone stimulates immune protection, increasing production of tumor-infiltrating lymphocytes (TIL) [100]. Besides, the antitumor effect of progesterone is provided through its regulatory action on the so-called long non-coding RNA, NEAT1/microRNA-146b-5p, which mediates the WNT/β-catenin signaling pathway [101]. It was shown that incubation with 20 μM progesterone significantly decreased expression level of the *NEAT1*, *miR-146b-5p*, *LEF1*, *c-myc*, and *MMP9* genes of the WNT/β-catenin signaling pathway in Ishikawa endometrial cancer cells, while the cell cycle was inhibited in the G0/G1 phase [101].

Another aspect of the possible clinical use of gestagens in oncology is their application as chemosensitizers of tumor cells, i.e. compounds increasing sensitivity of tumor cells to chemotherapy. Tumor cell resistance to the drugs used — the phenomenon of multiple drug resistance (MDR) — is known to be a significant limitation of anticancer therapy efficacy [102]. MDR is caused by overexpression of P-glycoprotein, multidrug resistance proteins (MRP), breast cancer resistance protein (BCRP), and other transmembrane transporters.

Four generations of P-glycoprotein inhibitors have been developed over the last 40 years; however, all these compounds have shown insufficient efficacy and high toxicity in clinical trials [103, 104].

Progesterone is an effective MDR modulator as most of the abovementioned transporter proteins have a specific progesterone-binding site [102]. It is known that progesterone regulates both the expression of P-glycoprotein mRNA and its protein levels [105], reduces BCRP-mediated MDR by suppressing BCRP expression in breast cancer cells, inhibiting transcription by binding to the progesterone promoter in the gene encoding BCRP [106]. It was shown in our paper that, in addition to their own cytostatic action on tumor cells, MPA, progesterone, and gestobutanoil gestagens exhibit chemosensitizing activity, increasing cytotoxic effects of cisplatin and etoposide by 20–50%, therefore, they can be used in combination with cytostatics at the first stages of chemotherapy [102].

Since one of the factors in the development of MDR is Wnt/β-catenin signaling pathway activation [107–109], the inhibitory effect of progesterone on this signaling pathway may also be a promising aspect in the clinical use of progestins as chemosensitizers.

## Conclusion

Gestagens are used in several areas of clinical practice — obstetrics and gynecology, oncology, in ART procedures, for cachexia and anorexia in cancer patients and AIDS patients.

Discovery of mitochondrial, membrane-associated, and membrane progesterone receptors, as well as in-depth investigation of their mechanisms of action in signaling pathways, contribute to obtaining new knowledge about the ways progestins exert their pharmacological effect. New data on progesterone regulation of MDR provide the possibility of using progestins as chemosensitizers in anticancer therapy and as cardio-, neuro-, and hepatoprotectors. Data on progesterone regulation of the immune system (immunosuppressive action) may contribute to widening the range of indications for administration of progestins, particularly, for combating viral and autoimmune diseases.

The role of progesterone in the body can hardly be overestimated because this sex hormone contributes to normal development of pregnancy, the most important physiological process in biology.
